# A study on the modulation of alpha-synuclein fibrillation by *Scutellaria pinnatifida* extracts and its neuroprotective properties

**DOI:** 10.1371/journal.pone.0184483

**Published:** 2017-09-28

**Authors:** Mahdyeh Sashourpour, Saber Zahri, Tayebeh Radjabian, Viktoria Ruf, Francisco Pan-Montojo, Dina Morshedi

**Affiliations:** 1 Department of Biology, Faculty of Science, Mohaghegh Ardabili University, Ardabil, Iran; 2 Department of Industrial and Environmental Biotechnology, National Institute of Genetic Engineering and Biotechnology (NIGEB), Tehran, Iran; 3 Department of Biology, Faculty of Basic Sciences, Shahed University, Tehran, Iran; 4 Center for Neuropathology and Prion Research, Ludwig-Maximilian University, Munich, Germany; 5 Department of Neurology, University Hospital, LMU, Munich, Germany; 6 Munich Cluster for Systems Neurology (SyNergy), Munich, Germany; Kermanshah University of Medical Sciences, ISLAMIC REPUBLIC OF IRAN

## Abstract

Aggregation of alpha-synuclein (α***-***SN) is a key pathogenic event in Parkinson’s disease (PD) leading to dopaminergic degeneration. The identification of natural compounds inhibiting α***-***SN aggregation may have a major role in treating PD. Different *Scutellaria* species are known as valuable medicinal plants, primarily due to their high flavonoid levels. *Scutellaria pinnatifida* (*S*. *pinnatifida*) is endemic to Iran; however, the knowledge of its pharmaceutical properties is limited. Here we report that *S*. *pinnatifida* extracts have an anti-fibrillation effect on α***-***SN aggregation and neuroprotective properties on PC12 and primary dopaminergic neurons. Treatment during α***-***SN fibril formation with *S*. *pinnatifida* extracts showed that the extractions performed with dichloromethane (DCMEx) and n-butanol (BuOHEx) strongly inhibited α***-***SN fibrillation. TLC-based analysis revealed that *S*. *pinnatifida* contains a great amount of flavonoids with high antioxidant properties as shown using a radical scavenging assay. Further analysis using HPLC and Mass spectroscopy on the DCMEx revealed the presence of baicalein in this extract. We then selected the more efficient extracts based on cell viability and ROS scavenging on PC12 cells and tested their neuroprotective properties on primary dopaminergic neurons. Our results showed the extracts strongly protected against α***-***SN oligomers. Surprisingly, they also neutralized the severe toxicity of paraquat. Therefore, *S*. *pinnatifida* may be a potential valuable medicinal herb for further studies related to the treatment of PD.

## Introduction

Parkinson’s disease (PD) is a chronic neurodegenerative disorder with severe medical and social impacts affecting more than 1% of people over the age of 65. If there is no access to accurate treatment, it is anticipated that the prevalence of this acute disease will be quickly expanded in the progressive ageing societies [1]. PD is mostly characterized by degeneration of dopaminergic neurons in the substantia nigra [2] leading to severe symptoms such as resting tremor, bradykinesia, muscular rigidity [3]. Strong evidence supports that aggregation of alpha-synuclein (α***-***SN) has a critical role in the pathogenesis of PD and other diseases, together known as synucleinopathies [4]. α***-***SN is a highly conserved protein, which is very abundant in the central nervous system (CNS) [5]. α***-***SN is a natively unfolded protein [6], changing its conformation into semi- helical structures by interaction with bio-membranes, especially negatively charged membranes. Under unknown conditions, α***-***SN tends to form amyloid fibrils with high ß-sheet contents [7]. Some aggregated species of α***-***SN formed along the fibrillation are very toxic and are able to intrude the functions of different organelles such as mitochondria, endoplasmic reticulum and plasma membrane [8–10]. Furthermore, it may increase the oxidative stress causing severe damages in dopaminergic cells [11,12]. It has been shown that some natural compounds like herbal flavonoids, including variable groups of polyphenols, are able to prevent aggregation and/or neurotoxicity of α***-***SN, and could thus potentially be useful to treat PD. Some well-known flavonoids such as baicalein, (-)-epigallocatechin-3-gallate (EGCG), scullcap flavones and wogonin were shown to influence diverse aspects of the neurodegenerative process, especially regarding α-SN aggregation. Most of these substances are strong inhibitors of α***-***SN oligomerization in both cell-free and cellular systems [13,14]. Some of them can even disaggregate and remodel preformed fibrils into monomers or nonpathogenic oligomers [15–18]. In this regard, it was recently shown that baicalein moderates some of the clinical symptoms induced by oral-rotenone in a PD mouse model and that this reduction is associated with a considerable reduction in the formation of α-SN oligomers [19]. The neuro-protective properties of EGCG have been confirmed in an MPTP induced mouse model of PD [20]. Furthermore, flavonoids are known to be radical-scavenging compounds that help to decrease oxidative stress [21]. The antioxidant activities of baicalein, scullcap flavones and wogonin have a close correlation with their neuroprotective effects [22,23]. Flavonoids also protect neuronal cells against the deleterious effects caused by inflammatory reactions [24,25]. It has been reported that these anti-inflammatory effects are due to their impact on the *NF-κB* pathway [26–28].

During the past decades, the use of herbal medicines as major natural factories of complex compounds has increased dramatically. In this regard, various species of the *Scutellaria* genus should be taken into consideration for pharmaceutical studies due to their remedial secondary metabolites, particularly flavonoids [22]. The genus of Scutellaria includes approximately 300 species. One of them, *Scutellaria baicalensis* is well known in Chinese traditional medicine and has been clinically used to treat allergies, hyper lipidemia, arteriosclerosis, and inflammatory diseases [29]. There is little information on the pharmaceutical properties of the Iranian species, *Scutellaria pinnatifida (S*. *pinnatifida*) [30–32]. It was determined that among 60 different Iranian herbs only 6 *S*. *pinnatifida* varieties have high anti-oxidant activity against linoleic acid peroxidation [31]. In another study, using the aerial parts of *S*. *pinnatifida*, it was shown that the methanolic extract (MeOHEx) had more antibacterial activity than the DCM extract (DCMEx), but DCMEx exhibited more antioxidant activity [32]. It was also indicated that its root CH_2_Cl_2_ extraction induced specifically apoptosis in some cancer cell lines [30]. However, its activity against α-SN fibrillation and its neurotoxicity or its protective effect against environmental toxins has never been investigated before. Therefore as a part of our ongoing research on the inhibition of α-SN amyloid fibrillation/ neurotoxicity, we screened the effect of different extracts from *S*. *pinnatifida* in these processes. In the current study, we find that S. *pinnatifida* inhibits amyloid fibrillation and protects against α-SN neurotoxicity. Furthermore, we also analyzed the antioxidant activity of the extracts and the neuroprotective effect against two well-known PD-related toxins, paraquat and rotenone, on primary dopaminergic neurons.

## Materials and methods

### Chemicals

Thioflavin-T (ThT), Congo red (CR), AlCl_3_·6H_2_O, acetic acid, 1, 1dipheny l-2-picrylhydrazyl radical (DPPH), paraquat, rotenone, baicalein were purchased from Sigma (USA). HPLC grade methanol, ethanol, n-hexane, dichloromethane, ethyl acetate, n-butanol, di-phenyl boric acid ethanol amine and polyethylene glycol were obtained from Merck (Germany). Other compounds were also purchased from Merck (Germany).

### Expression and purification of α-SN

**Fig 1:**
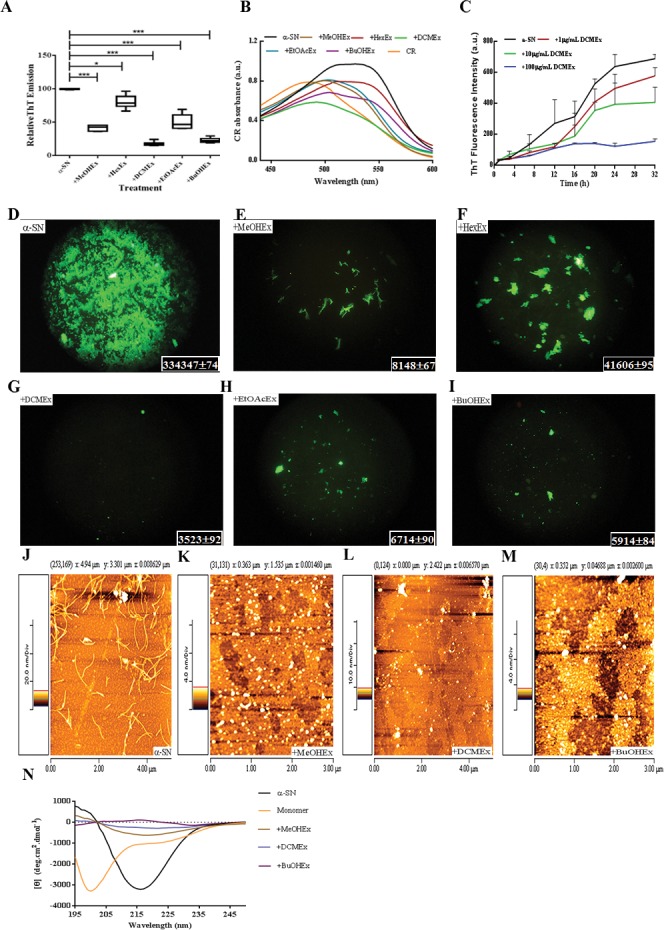
Effects of different extracts of *S*. *pinnatifida* on α-SN fibrillation. (A) ThT fluorescence intensity; and (B) CR absorbance measurements of α-SN alone and in the presence of MeOHEx, HexEx, DCMEx, EtOAcEx, and BuOHEx. The spectrum of CR alone is also shown. (C) Kinetic analysis of DCMEx effect on the fibrillation of α-SN at different concentrations (1, 10 and 100 μg/mL) monitored by ThT fluorescence emission at 488nm. (D-I) Fluorescence microscopy images; Images include of protein aggregates untreated and treated with the extracts. By using Image J, analysis of the fluorescence particles’ density present in each image has been carried out and shown in the right bottom of the image. (J-M) AFM images of α-SN aggregates in the absence and the presence of the selected extracts after incubation for 24 h. (N) CD spectra of monomeric and fibril forms of α-SN in the absence and presence of the selected extracts of *S*. *pinnatifida*. Error bars = SD, n = 3, * represents P < 0.05, and *** represents P < 0.005.

Recombinant human α-SN-containing pNIC28-Bsa4 plasmid was transformed into *Escherichia coli* BL21 (DE3) pLysS cells (Novagen, Madison, WI, USA). Expression was induced with IPTG and α***-***SN was extracted and purified according to Huang et al [33]. Briefly, the purification of α-SN was carried out in three steps, including osmotic shock, anion-exchange and size exclusion chromatography. Finally, the sample was purified using an Amicon Ultra Centrifugal Filter (10kDa). The concentration of the purified protein was determined using the Pierce BCA Protein Assay Kit and samples were freeze-dried and stored at -20°C in aliquots. The final purified α-SN was tested with native-PAGE and SDS-PAGE, and also with Western blotting using specific monoclonal antibody against α-SN (data not shown). Far-CD spectroscopy also indicated the random coil structure for the purified protein in its monomeric form (Fig 1N).

### Plant materials and extraction

The root of *S*. *pinnatifida* was collected in Jun 2014 from the Damash area. Damash is a village in Jirandeh Rural District, Amarlu District, Rudbar County, Gilan Province, Iran with geographic coordinates of 36.7566° N, 49.8100° E and is out of the protected areas at the north part of Iran. The plant was identified by Dr. Attar at the Herbarium of Tehran University (Tehran, Iran) where a voucher specimen (herbarium no: 45950-TUH) has been deposited. No permissions for collecting the plant were obtained because the plant was not in the list of endangered plants of Iran. The name of this plant is not mentioned in the IUCN protection list (http://www.iucnredlist.org/). Also in the book entitled “a preliminary survey of endemic, rare and endangered plant species of Iran", that introduced endangered plants of Iran, there are no mention of this plant. It is in LC category and is not endangered according to the herbarium book code IRAN. Furthermore, the plant grows in the wide extends of the North, North East and West of Iran. In order to prepare various extracts from *S*. *pinnatifida*, the root was carefully washed with water and dried under shade at room temperature. The dried plant root was powdered using an electric blender (Bosch MKM6003, Germany). About 10 g of the powdered material was extracted with a mixture of methanol and water with a 9:1 ratio (v/v) using a bath sonicator (Badelin SONOREX Digital 10P). Samples were sonicated 3 x 15 min. After filtrating with a Boehner funnel, the filtrated solution was centrifuged (3000 rpm, 5 min) to remove small suspended particles. The lyophilized (labogeen ScanCool, Denmark) methanolic extract (MeOHEx) was sub-extracted with n-hexane (HexEx), dichloromethane (DCMEx), ethyl acetate (EtOAcEx) and n-butanol (BuOHEx) serially, based on a two-phase solvent system. In this process, 0.3 g of lyophilized MeOHEx was distributed in 10 mL water. The water-distributed sample was sub-extracted with an equal volume of the immiscible solvents. Each extract was lyophilized again and re-suspended in a solvent of DMSO and buffer or DMSO and culture media with a ratio of 1 to 10 (v/v). In the next step 10μL of each re-suspended extract solution was added to 90 μL of the subjected samples in which the finally concentration of the extract was 100 μg/mL. The concentration of DMSO in the subjected samples was 1% (v/v). Effects of DMSO on the fibrillation/cytotoxicity of α-SN were also assessed.

### Induction of α-SN fibrillation and treatment with the extracts

140 μM of recombinant α***-***SN protein was dissolved in 1 mL of TNE buffer (30 mM Tris-HCl, 1 mM EDTA and 0.1 mM NaNO_3_, pH 7.5) and then incubated at 37°C for 24 h with a fixed shaking speed (85 rpm) in a shaking water bath (Thermo Scientific HAAKE Shaking Water Bath (SWB25)) to allow fibril formation. To assess whether the different extracts of *S*. *pinnatifida* could inhibit α***-***SN aggregation, the same reaction was performed in the presence of the extracts in DMSO as the final solvent. In each treated sample 1 to 10 v/v of the re-suspended extract was added in which the final concentration ratio of the protein to extract was 20:1 w/w. As control some samples were treated with the same volume of DMSO.

#### Generation of α-SN oligomers

Generation of α*-*SN oligomers for the experiments with dopaminergic neurons was done as follows. α-SN was fluorescently labeled with an amino reactive red fluorescent dye (Alexa Fluor-647) as described previously [34]. Fluorescently labeled α-SN and unlabeled α-SN were mixed at a ratio of 1:5. Hereupon, oligomer formation was induced at a concentration of 50 μM α-SN in the presence of 100 μM Al^3+^ under constant shaking (1400 rpm) at 37°C for 30 minutes and confirmed by single molecule fluorescence spectroscopy (S1 Fig). Oligomers were stored at -80°C. Oligomer formation was verified by single molecule fluorescence spectroscopy.

### Assessment of the fibril formation

#### ThT assay

α*-*SN aggregation was monitored using ThT fluorescence by using a 500 μL tris (pH 7.4, 50 mM) solution containing 1.5 μM α-SN, and 20 μM ThT. The fluorescence intensity was measured at room temperature using a Varian Cary Eclipse fluorescence spectrophotometer (Mulgrave, Australia) and setting the excitation and emission wavelengths at 440 nm and 450–550 nm, respectively.

#### Congo red (CR) absorbance assay

Formation of fibrillar aggregates was also assayed using a fresh CR solution. CR was dissolved at a final concentration of 1 mg/mL in a buffer containing 150 mM of NaCl and 5 mM of Na_2_PO_4_ (pH 7.4). A 10 μL of the incubated sample was added to 490 μL of the CR solution and incubated for 5 min. Absorbance spectra were measured from 400 to 600 nm by the PGT80+UV–Visible spectrometer (Leicestershire, England) [35]. In some cases, interaction of CR with some forms of the aggregates (especially fibrillar forms) resulted in red shifting of the position of the absorbance maxima. The way to quantify red shifting is through the spectral center of mass (υ_g_) [36]. υ_g_ is defined based on Eq (1):

$$\nu_{g}=\frac{\sum F_{i\nu_{i}}}{\sum F_{i}}$$ Eq (1)

Where F_i_ is the emission at a wavenumber, υ_i_, and the sum is carried over all wavenumbers where F_i_ > 0.

#### Fluorescence microscopy analysis

15 μL of the incubated protein were added to a 15 μL of ThT solution (with 15 μM of ThT). The mixture was incubated at room temperature for 5 min and after spreading onto a microscopic slide, it was studied by a fluorescence microscopy (Ceti Inverso TC100 microscope, Medline scientific, Oxon, UK). Image analysis was performed via Image-J 1.44 P. The area occupied by the fluorescent particles was compared between different samples by adjusting color threshold parameters in fixed numbers (Hue: 45–117, Saturation: 100–255, Brightness: 100–255).

#### Circular Dichroism (CD) spectropolarimetry

Far-UV CD spectropolarimeter was used to analyze the secondary structure of α*-*SN. Using an AVIV 215 CD-spectropolarimeter, we analyzed structural changes induced in α*-*SN in the presence of the plant extracts. To conduct these experiments, 10 times diluted α*-*SN samples, treated with MeOHEx, DCMEx and BuOHEx were prepared and spectral properties of the incubated protein were monitored using a 0.1 cm diameter cell. CD spectra of TNE buffer with or without 1% resuspended extracts (in DMSO (was recorded and subtracted from the protein spectra and the CD signal given as ellipticity.

#### Atomic Force Microscopy (AFM) imaging

The incubated α-SN in a fibril formation condition was diluted 20 times with deionized water, then a small aliquot (10 μL) was deposited on a freshly cleaved mica sheet. The treated mica sheet was allowed to be air- dried. AFM was performed at room temperature by a NanoScope IIId controller from Veeco Instruments Co. (Plainview, NY, USA) with a silicon probe (CP-CONT-PM, sphere without coating). Imaging was performed under the tapping modality.

### Assessment of the antioxidant activity of the extracts

The antioxidant activity of the extracts was evaluated using 2, 2-Diphenyl-1- picrylhydrazhyl (DPPH) as previously described by Kim et al [37].

Briefly, a methanolic solution of DPPH (0.002% w/v) was prepared. 1 mL of DPPH was mixed with 1 mL of the crud extract or sub-extracts of *S*. *pinnatifida*. The absorbance was monitored at 517 nm after 30 min of incubation at room temperature and in the dark using a PGT80+UV–Visible spectrometer (Leicestershire, England). The antioxidant activity (AA) was calculated using Eq (2):

$$AA(\%)=\frac{\left(A_{0}-A_{s}\right)}{A_{0}*100}$$ Eq (2)

where A_O_ is the absorbance of the blank (1 mL of 0.002% (w/v) DPPH + equal volume of methanol) and A_S_ is the absorbance of the samples.

### Determination of total flavonoids content

The total flavonoids content of *S*. *pinnatifida* extracts were determined using the AlCl_3_ method [38]. This method is based on the formation of a flavonoid-aluminum complex. 100 μL of the extract was added to an equal volume of a 2% AlCl_3_·6H_2_O solution (2 g AlCl_3_·6H_2_O per 100 mL methanol). After shaking vigorously, the sample was incubated at room temperature for 10 min. Then the absorbance was measured at 430 nm with a UV-Vis spectrophotometer using a PGT80+UV–Visible spectrometer (Leicestershire, England). Quantitative determination of total flavonoids was done based on the standard curve of baicalein. The contents of the flavonoid compounds were expressed as milligram baicalein equivalents to gram of dry weight.

TLC was also performed in order to detect total flavonoid compounds in the plant extracts [39]. 30 μL of the extracts were spotted on the silica gel G60 F254-pre-coated TLC plates (10× 20 cm). The plates were then placed in a chromatography chamber and saturated with a mobile phase solvent (n-butanol–acetic acid- water, 10:1:5, v/ v/ v) at room temperature.

The developed plate was air-dried and then sprayed with a 1% solution of the flavonoid-specific reagent, diphenylboric acid ethanolamine in methanol. After drying with 5% polyethylene glycol, the presence of flavonoids was visualized under UV light at 366 nm in a camera equipped chamber [40].

#### HPLC and Mass spectroscopy (MS) analysis

To assess of the presentation of baicalein in DCMEx, HPLC and Mass spectroscopy (MS) analysis were carried out using pure baicalein (Sigma-Aldrich, 11712) as a standard. The HPLC apparatus was a Smartline model (Kenuer, Germany) with a quaternary pump and a reversed phase column C18 Eurospher-100 (5 μm particle, 125 mm × 4 mm) coupled with a UV-VIS detector (D-14163 model). Data were processed by Software ChromGate (version 3.1). Mobile phases consisted of water with 0.2% glacial acetic acid (solvent A) and acetonitrile (solvent B) in gradient mode. Initial condition was A–B (90:10, v/v), linearly changed to A–B (35:65, v/v) after 20 min. and holded at the same ratio for 10 min. Then the percentage of mobile-phase A increased to 90% after 35 min and reached 0% after 45 min. The flow rate was kept at 1 mL/min. The injection volume was 20 μL, and peaks were monitored at 277 nm. Samples were filtered through a hydrophilic PTFE membrane filter with a 0.45 μm pore size before injection. Peak of baicalein was identified by congruent retention time.

MS analysis of baicalein was conducted on an Agilent 6410 Triple Quadruple mass spectrometer, coupled to an Agilent 1200 series liquid chromatography equipped with an auto sampler (1200 series) and a diode array detector (1200 series). Experiments were carried out with an ESI source in a positive ion mode. The fragmentor was set at 80 and the collision energy on 35v. The solvent program was set as a flow rate of 0.5 mL/min with 90% Methanol and 10% water contain 0.1% formic acid. The source was operated using 300°C drying gas (N2) at 6 L/min, nebulizing gas adjusted at 10 psi and capillary voltage adjusted at 4000V. Multiple reaction monitoring (MRM) detection was employed using nitrogen as the collision gas, with a dwell time of 150 ms for each transition; the transition monitored for baicalein was m/z 271→123.

### Culture of PC12 cells and primary mesencephalic dopaminergic neurons

PC12 cells were cultured in Dulbecco’s Modified Eagle’s Medium (high glucose) supplemented with 10% fetal bovine serum, 100 units/mL penicillin (Pen), and 100 μg/mL streptomycin (Strep). The cells were incubated in a humidified incubator at 37°C under an atmosphere containing 5% CO_2_.

All animal procedures were performed according to the German Law for Animal Experiments (Tierversuchsgesetzt) and were approved by the Regierung von Oberbayern (The government from Bayern, Germany). Primary mesencephalic neuronal cell cultures were prepared as previously described [41]. Briefly, E14.5 embryos were obtained from C57JBL6 pregnant mice after cervical dislocation. Brain mesencephali were dissected under the microscope and digested with Trypsin-EDTA 0.12% (Life Technologies, USA) for 7 min. The trypsin reaction was then stopped by adding basic medium (BM), containing Neurobasal A medium (Gibco), 1 mg/mL Pen/Strep, 10% FCS and 200 mM L-Glutamine, and cells were mechanically dissociated using a fire-polished Pasteur pipette. Medium was fully replaced after 5 min, centrifugation at 1200 rpm, aspiring the supernatant and adding 8 mL of fresh BM to the pellet. Concentration of cells in the medium was estimated using a Neubauer chamber and a 100 μL of medium containing 10^6^ cells /mL plated per well in a 96-well plate (Greiner Sensoplate, Germany). Then a 20 μL of medium was removed from the well and 24 h later 1/3 of the media was replaced with fresh BM. On DIV3 half of the medium was replaced with B27 one, containing Neurobasal A medium, 1mg/mL Pen/Strep, 200 mM L-Glutamine and B-27 supplement; and on DIV5 all medium was replaced by B27 medium. Treatment was administered on DIV7 and DIV 9 cell and cells were fixed on DIV10.

To determine the optimal concentration of the plant extracts to be used on the dopaminergic neurons, we tested different extract concentrations, including 1, 10 and 100μg/mL, on the cultured neurons.

#### Cell toxicity assessment on PC12 cells and dopaminergic neurons

The effect of the plant extracts on the α-SN-induced cell death on PC12 cells was estimated by measuring MTT reduction. This assay is based on the conversion of the yellow tetrazolium salt (MTT) to the purple formazan by mitochondrial dehydrogenase of live cells [42]. Briefly, PC12 cells (3×10^4^ cells/200μL/well) were added to the wells of 96-well plates. To study the cytotoxic effects of α-SN in the presence and absence of the extracts, a 10% (v/v) dilution of fibrillated α-SN, incubated for 24 hours in the presence or absence of MeOHEx, DCMEx and BuOHEx extracts, or just the extracts as controls were added to PC12 cell cultures for 24 h. After treatment, culture medium was replaced with new medium and MTT solution was added to the cells (with a final concentration of 0.5 mg/mL). Plates were incubated at 37°C for 4 h. The solution was then removed, and the precipitated formazan crystals were solubilized in a 100 μL DMSO. Absorbance was measured at 570 nm using a plate reader (Expert 96, AsysHitch, Ec Austria).

To further characterize cytotoxicity of α-SN and the protective effect of the extracts, the proportion of apoptotic and necrotic PC12 cells, Annexin V and Propidium iodide (PI) positive respectively, was determined by flow cytometry. For this, PC12 cells were seeded on a 6-well microtiter plate (5x10^5^cells/well) and treatment as described above was performed on DIV1. At the end of the incubation period, PC12 cells were harvested, washed with cold PBS and resuspended in 500 μL of the binding buffer. The cells were double stained with the fluorescein isothiocyanate (FITC)-conjugated Annexin and PI, according to the instructions of the kit (The FITC Annexin V/PI Dead Cell Apoptosis Kit, Invitrogen), kept at a dark place for 15 min just before analysis. Samples were then loaded on the BD FACSCalibur flow cytometer (Becton Dickinson, Franklin, Lakes, NJ, USA) to analyze the proportion of different kinds of dead cells. Flowing software v.2.5 was then used to discriminate early or late apoptosis.

The protective effect of the DCMEx and BuOHEx/EtOAcEx (in a ratio of 1:1 (w /w)) against α-SN oligomers-, paraquat- and rotenone-induced cell death on dopaminergic neurons was assessed through manual counting of immunostained TH^+^ neurons after treatment. Briefly, neurons were prepared and plated on 96 well plates as described above. On DIV7, the neurons were treated with 10 μM α-SN, 10 nM rotenone and 12.5 μM paraquat alone or together with 1μg/mL of DCMEx or 100μg/mL BuOHEx/EtOAcEx for three days. After this time, cells were life stained using DCFH-DA to perform the intracellular Reactive Oxygen Species (ROS) assay (see section 2.12) or were fixed using 4% paraformaldehyde for immunocytology (see section 2.11).

Dopaminergic TH+ neurons were observed using an inverted fluorescence microscope (Olympus) under a 20x objective. The diameter of every well was scanned in two perpendicular directions (i.e. top to bottom and left to right) and total TH+ neurons were counted for every well.

#### Immunocytology of mesencephalic cell cultures

4% PFA fixed neuronal cell cultures were washed 3x10 min in phosphate buffered saline (PBS), blocked using a blocking solution (BS) (0,2% Triton X-100 in PBS and 5% donkey serum (DS)) for 1 h at room temperature, and incubated with a mouse anti-TH (Clone LNC1,1:500, Millipore, MAB318) primary antibody in BS overnight at 4°C. On the next-day cells were washed 4x10 min with PBS, incubated with a donkey Alexa® 555 anti-mouse secondary antibodies for 1 h at room temperature and washed 4x10 min with PBS.

#### Intracellular ROS assay

The level of intracellular ROS in PC12 and primary mesencephalic neuronal cultures was measured using a fluorogenic and plasma membrane permeant dye, DCFH-DA (2, 7-dichlorofluorescein diacetate). After diffusing into the cell, DCFH-DA is deacetylated enzymatically and later oxidized by ROS into a fluorophore compound, 2’, 7’–dichlorofluorescein. Briefly, PC12 cells were seeded into 96-well plates (3.0x10^4^ cells /well/200μL) and cultured for 24 h. On the next day, cells were treated with 20μL of 7 h-aged fibrillated α*-*SN alone or in combination with the extracts. Mesencephalic neurons on the other side were treated as previously described. In both cases, after treatment, cells were washed one (mesencephalic neurons) or two (PC12 cells) times with FBS free DMEM and incubated with 20 μM DCFH-DA at 37°C for 30 min. Extra DCFH-DA was removed by washing the wells with phosphate-buffered saline (PBS, 0.01 M, pH 7.4). Fluorescence intensity was measured using 480 nm excitation and 520 nm emission with a Varian Cary Eclipse fluorescent spectrophotometer (Mulgrave, Australia) for PC12 cells and a Fluostar OPTIMA (BMG Labtech, Germany) for mesencephalic neurons.

## Results

### Extracts obtained from ground root of *S*. *pinnatifida* have strong inhibitory effects against α*-*SN fibrillation

To analyze the effects of *S*. *pinnatifida* root extracts on α-SN fibrillation, its fine- ground root was dissolved in a 90% methanol solvent and sonicated. Subsequently, the lyophilized methanolic extract (MeOHEx) was sub-extracted with n-hexane (HexEx), dichloromethane (DCMEx), ethyl acetate (EtOAcEx) and n-butanol (BuOHEx) serially, based on a two-phase solvent system. The purified recombinant α***-***SN protein was incubated under fibrillating conditions in the absence or presence of the different extracts of S. *pinnatifida* root. To examine the effects of the *S*. *pinnatifida* extracts, we used ThT to assess fibril formation. In the presence of some extracts, especially DCMEx and BuOHEx the fibril formation of α-SN after 24h significantly decreased (see Fig 1A).

The result of CR absorbance assay is shown in Fig 1B. When amyloid fibrils interact with CR, they induce a characteristic increase in absorption and a red shift in the absorption maximum. The analysis data of the spectral center of mass (Eq (1)) has been presented in S1 Table.

In the presence of incubated α-SN, the CR’s center of mass shifts from 497.7 to 514.2 nm. Treatment of α-SN with HexEx had almost no effect on the shifting. However, in the present of the other extracts the CR’s center of mass showed a 50% reduction in the red-shift. Nevertheless, we observed some changes in the pattern of CR absorbance when CR was added to the pre-treated α-SN. In these cases other aggregated forms of the protein may interact with CR and change the absorbance spectrum of CR. It is established that CR can interact with other structures apart from amyloids [43].

To analyze in more detail the inhibitory activity of DCMEx on α-SN fibrillation, the kinetic of α-SN fibrillation was investigated in the presence of 1, 10 and 100 μg/mL of DCMEx. As shown in Fig 1C, the activity of DCMEx against fibrillation is dose dependent and at the low concentration, 1μg/mL, DCMEx did not show any considerable inhibitory activity. In spite of this result, we will show that the presence of 0.1 μg /mL DCMEx in the media has a protective activity on the treated cell against the toxic oligomeric forms of α-SN.

As the fluorescence emission of ThT is significantly increased when ThT interacts with the fibrillar forms of α***-***SN, it was used in fluorescence imaging study [44]. As shown in Fig 1D–1I, we observed differences in the density of fluorescent particles in the different treatments. The density of the fluorescent particles in each sample was calculated and the data was shown in each image in a box.

We then investigated whether the treatment with the root extracts modulated the morphology of α***-***SN aggregates using AFM. As shown in Fig 1J–1M, typical fibrils were abundantly found in the untreated sample (J) but in the samples treated with the MeOHEx (K), DCMEx (L), or BuOHEx (M) just non-fibrillar particles were formed.

CD analyses were carried out to qualitatively assess the structural changes induced on α***-***SN protein by the presence of the *S*. *pinnatifida* extracts. Fig 1N illustrates the far-UV CD spectra α***-***SN after 24 h of incubation in the presence and absence of the selected extracts compared to the monomeric form of the protein. After incubation, α***-***SN had a strong negative peak approximately at 218 nm, indicating beta sheet structures. The CD spectrum analysis of the monomeric form of α***-***SN showed a random coil structure with a sharp negative peak around 200 nm. The samples treated with the extracts did not exhibit visible peaks around 200 or 218 nm, suggesting that, although the native monomeric form was not stabilized, protein aggregates’ species did not have high contents of beta sheet structures.

### *S*. *pinnatifida* extracts have radical scavenging activity and high levels of flavonoids

It is believed that oxidative stress plays a major role in pathogenesis of neurodegenerative diseases, especially PD [45]. To monitor the antioxidant activity of the extracts, DPPH dye assay was carried out. As indicated in Table 1, most extracts except HexEx exhibit a strong antioxidant activity with able to neutralize DPPH radicals.

**Table 1:** Evaluation of the free-radical scavenging activities and total flavonoids content of the different extractions of *S. pinnatifida* using DPPH and AlCl_3_ methods.

Extract	Antioxidant activity^a^ (%)	Flavonoid content^b^ (mg/g of the dried extract)
MeOHEx	92.17 ±0.17	1.51±0.06
HexEx	49.28±0.10	0.01±0.00
DCMEx	90.86 ±1.88	2.24±0.03
EtOAcEx	90.08 ±0.70	1.01±0.01
BuOHEx	96.02 ±1.90	1.42±0.01

^a^ by using DPPH method after 30 min incubation and comparing with the standard sample using Eq (2).

^b^ by using Al_3_Cl method

Data represents Mean ± SEM of three independent experiments

We assumed that this antioxidant activity was due to the flavonoid content of the extracts. We therefore measured the total flavonoid content of the crude extract and the sub-extracts using AlCl_3_ (Table 1).

The results of the AlCl_3_ assay showed that different levels of flavonoids were present in the analyzed extracts. MeOHEx, DCMEx, and BuOHEx presented high level of flavonoids. In contrast HexEx contained considerably lower level of flavonoids. By using a specific dye for flavonoids in the TLC (diphenylboric acid ethanolamine) we also found with a different method that MeOHEx, DCMEx, and BuOHEx contain flavonoids. Interestingly, when compared to a standard of Baicalein (a well-known flavonoid) that was also loaded on the TLC, the DCMEx showed a strong wide band at the same height like the baicalein band. A similar result was obtained through HPLC analysis of the DCMEx, showing a peak with a retention time of 13 min., similar to the one observed when analyzing the baicalein standard with the chromatogram (S2 Fig).

In order to determine more detail of the extract, we also performed a MS analysis [46], which confirmed the presence of baicalein in the sample. The MS analysis was performed on the positive ion mode. The electrospray ionization of baicalein produced protonated molecular ions at m/z 271.4. Based on the major product ions in the product ion mass spectrum, MRM transition was selected m/z 271.4→123.1 for baicalein (S3 Fig).

### Protective effects of *S*. *pinnatifida* extracts on PC12 cells against the cytotoxicity of α-SN

To evaluate the toxicity of α-SN on PC12 cells and the protective effect of *S*. *pinnatifida* extracts, we used the MTT assay on PC12 cells after incubation in the presence of α***-***SN fibrillated alone or fibrillated in the presence of MeOHEx, DCMEx, or BuOHEx. First we tested the toxicity of the extracts alone. At the concentrations in which DCMEx had the highest significant effect on α-SN aggregation (100 μg/mL, final concentration in PC12 media of 10 μg/mL) it also showed high neurotoxicity (S4A Fig). However, we observed no toxicity when 1 μg/mL of the extract was present in the media, which corresponded to adding 10 μg/mL to α-SN during fibrillation.

**Fig 2:**
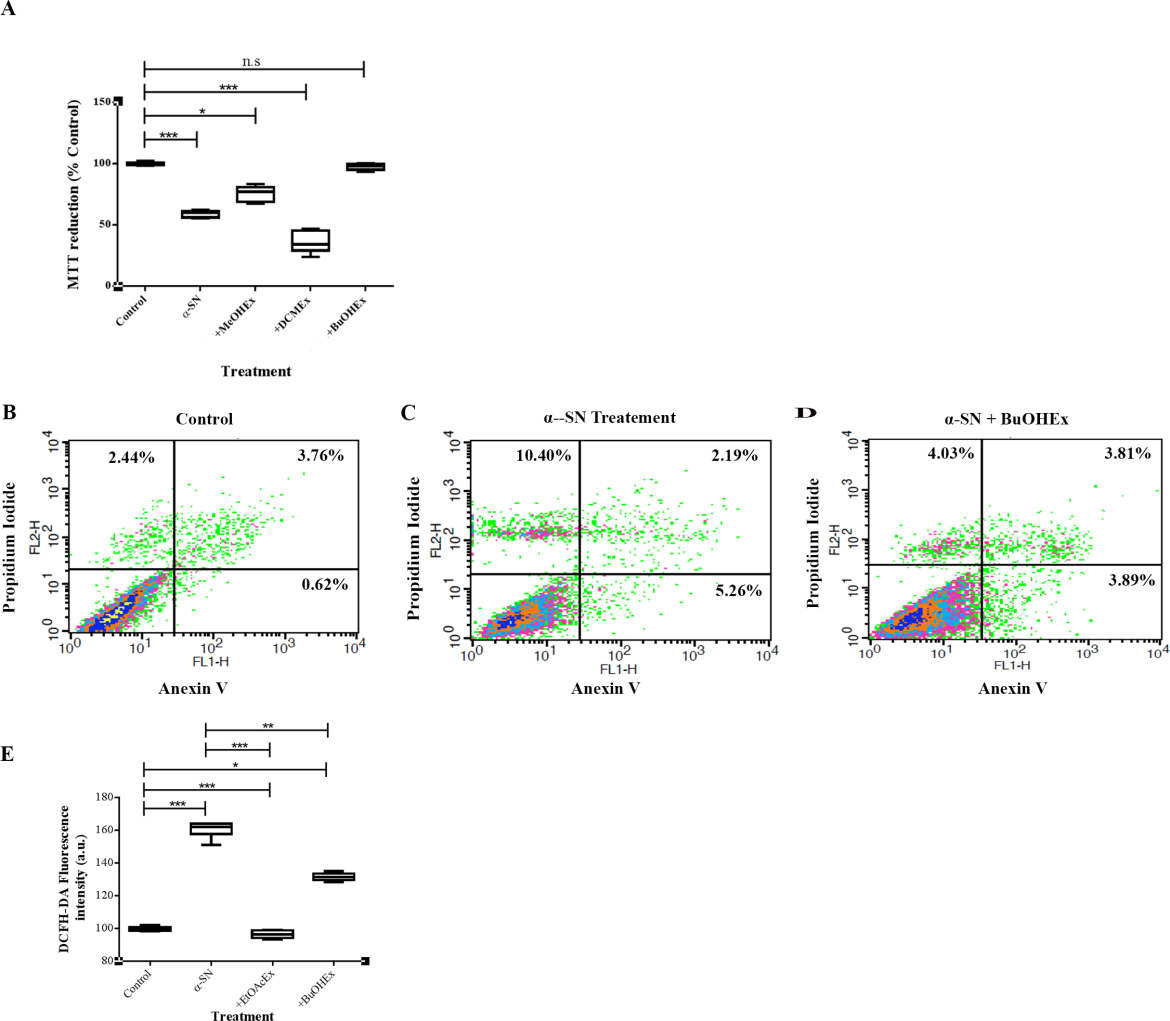
Assessment the cytotoxicity of α-SN on PC12 cells in the presence of the extracts. (**A**) Cell viability measuring by MTT assay. PC12 cells were treated with 7 h-aged incubated α-SN in the absence and presence of different extracts. (**B-D**) Analysis of the cell death type using Annexin V/PI method: Living cell (Annexin V−/PI−) populations were located in the lower-left quadrant, the apoptotic cells were in the lower-right quadrant, late apoptotic (Annexin V+/PI+) populations were located in the upper-right quadrant, and necrotic cell (Annexin V−/PI+) populations were presented in the upper-left quadrant.(**E**) ROS production assay. Fluorescence intensity was measured at 480nm excitation and 520nm emission. The significance was set at *P*<0.05.* The data are the means of three independent experiments ± SEM.

No remarkable cytotoxic activity was observed when the cells were treated with 100 μg/mL of MeOHEx or BuOHEx (S4A Fig). Our results show that short-term incubated α-SN (7 h incubated in fibrillating conditions) had a significant lethal effect on PC12 cells (48% of cell death) in comparison with the untreated cultured cells. Adding the protein pre-incubated with 100 μg/mL BuOHEx (10 μg/mL final concentration in the media) and with 10 μg/mL DCMEx (1 μg/mL final concentration in the media) increased the viability of PC12 cells when compared to untreated α-SN (Fig 2A).

In contrast, the addition of α-SN pretreated with 100 μg/mL of DCMEx (10 μg/mL final concentration in the media) lead to a significant increase in cytotoxicity when compared to treatment with α-SN alone, probably due to the toxicity of the extract in this concentration.

Annexin V/PI staining showed an increase in the number of apoptotic cells in the presence of 7h-aged amyloid fibrils (Fig 2C). Pretreatment and the presence of BuOHEx with α-SN also reduced the apoptotic rate (Fig 2D).

We also analyzed the antioxidant properties of *S*. *pinnatifida* extracts at the cellular level. Staining with DCFH-DA showed that treatment with 7h-aged α***-***SN increased the intracellular ROS production (63%). However, pretreatment of α-SN with EtOAcEx or BuOHEx during the incubation period and their presence in the cell culture significantly reduced ROS production (Fig 2E).

### *S*. *pinnatifida* extracts protect dopaminergic neurons against α-SN oligomers and paraquat toxicity

**Fig 3:**
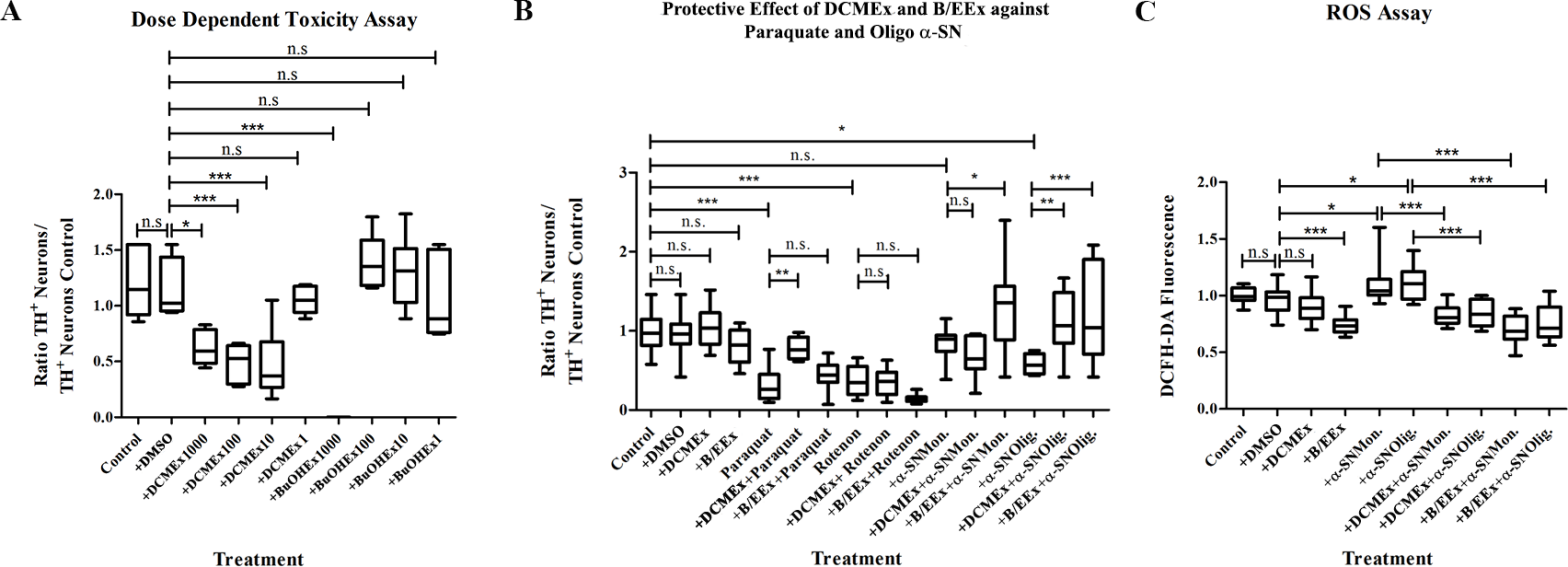
*S*. *pinnatifida* extracts are neuroprotective against paraquat and oligomeric α-SN toxicity on dopaminergic neurons in primary mesencephalic cell cultures. (**A**) Box-Plot Graphic showing the effect of 1, 10, 100 and 1000 μg/ml to determine the non-toxic concentration of DCMEx (DCM) and BuOHEx / EtOAcEx (BE) on dopaminergic TH^+^ neurons. (**B**) Box Plot Graphic showing the toxic effect of paraquat, rotenone and an α-SN oligomer/monomer mixture on TH^+^ neurons and the protective effect of 100 μg/mL BuOHEx / EtOAcEx (BE) or 1 μg/mL DCMEx (DCM) against this aggression. (**C**) Box-Plot Graphic showing the effect of α-SN oligomers on ROS production in mesencephalic cells and its reduction in the presence of DCMEx, and BuOHEx / EtOAcEx. Whiskers represent Max and Min values. * represents *P* < 0.05, ** represents *P* < 0.01, n.s. non-significant.

**Fig 4:**
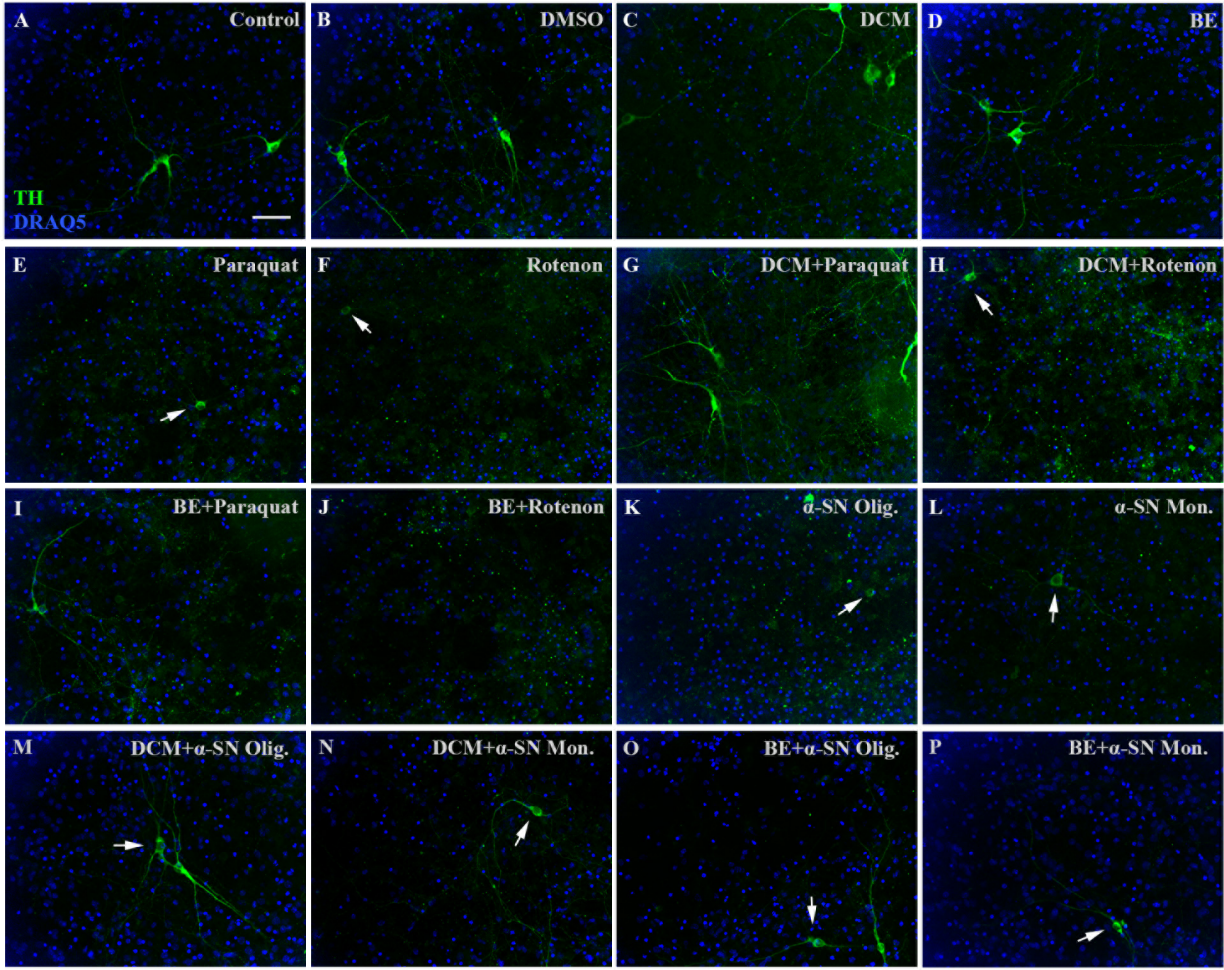
Fluorescence microscopy images of mesencephalic neurons. **(A-P).** Microscope images from immunostained mesencephalic neuronal cultures. Scale bar 20 μm. Cells were fixed and stained against tyrosine hydroxylase (TH, green). Nuclei were then stained with DRAQ5 (blue). TH^+^ neurons treated with DMSO (**B**), DCMEx (**C**) and BuOHEx / EtOAcEx (**D**) showed no morphological alterations when compared to control (**A**). Treatment with Paraquat (**E)**, Rotenone (**F**) and an α-SN-oligomer-monomer mixture (**G**) induced morphological alterations in the form of neurite loss (arrows in **E-G**) and the previously described neuronal loss. This effect was not so prominent when α-SN-monomers were used for treatment. DCMEx and BuOHEx/EtOAcEx co-treatment was protective against paraquat (**I** and **M**) and α-SN-oligomer (**K** and **O**) toxicity. However, it was not able to rescue rotenone toxicity or its morphological phenotype (**J** and **N**).

We wanted to analyze the neuroprotective effect of DCMEx on dopaminergic mesencephalic murine neurons. Based on the DPPH results, we also treated the cells with a mixture of EtOAc and BuOH to combine the antioxidant and neuro-protective activity of both compounds. In order to mimic the pathophysiological process and investigate the intracellular neuroprotective properties of the extracts, we did not induce α***-***SN oligomer formation in the presence of the extracts, but these were only added to the medium together with α***-***SN treatment. First, we determined the optimal treatment concentration of both extracts. For this, we treated dopaminergic neurons with different concentrations of the extracts (1, 10, 100, 1000 μg/mL) and counted the amount of TH^+^ neurons after the treatment. Whereas for DCMEx concentrations higher than 1 μM were toxic to TH^+^ neurons, in the case of BuOHEx/EtOAcEx concentrations up to 100 μM showed no toxic effects (Fig 3A). We then analyzed the neuroprotective effect of both extracts by treating dopaminergic neurons with α-SN monomers, an α-SN oligomers-monomer mixture, rotenone and paraquat alone or in combination with 100 μg/mL BuOHEx/EtOAcEx (in a ratio of 1/1(w/w) or 1 μg/mL DCMEx. Treatment with 12, 5 μM of paraquat, 10 nM of rotenone or 10 μM of the α-SN oligomer-monomer mixture induced morphological alterations and the loss of TH^+^ neurons (Fig 4A–4P). Our results show that BuOHEx/EtOAcEx and also DCMEx protected TH^+^ neurons against paraquat- and α-SN oligomers induced toxicity (Fig 3B). However, no significant protection against rotenone was observed (Fig 3B).

Finally, we also analyzed the anti-oxidant effect of both extracts on mesencephalic neurons. Our results show 100 μg/mL BuOHEx/EtOAcEx or 1 μg/mL DCMEx alone reduced ROS production. This effect could also be observed in the presence of α-SN oligomers (Fig 3C).

## Discussion

This study examined the neuroprotective effects of *S*. *pinnatifida* extracts through their effects on α***-***SN fibril formation and cytotoxicity. Since the medicinal properties of various species of the *Scutellaria* genus have been verified in many studies [22,47,48] and the presence of two flavones, skullcap flavone II and wogonin, have been detected in the root of *S*. *pinnatifida* [49], we hypothesized that fibrillation and also cytotoxicity of α***-***SN could be modulated by *S*. *pinnatifida* extracts.

In order to achieve efficient extraction compounds from the dried root of *S*. *pinnatifida*, in the first step a methanolic extraction and in the second step two-phase solvent extraction system were employed. To achieve extracts with high contents of α-SN fibrillation/cytotoxicity inhibitors along with ROS scavenging activity.

In this study, assessment of α-SN fibril formation using different methods showed that among different extracts, DCMEx and BuOHEx possessed the highest inhibitory activity against α-SN fibrillation. The CD and AFM data indicated that co-treatment of α-SN with the extracts reduced the content of fibrillar beta sheet structures. Thus, suggesting that in the presence of the extracts, the pathway for formation of high ordered fibrils with β-sheet structure was inhibited and small non-fibrillar particles were produced. Different studies on the mechanism of α-SN aggregation have shown that a common conformation exists between the toxic oligomers of α-synuclein and other aggregate-forming proteins and that this involves a high amount of β-sheet secondary structure that increases the protein-cellular membrane interactions leading to cytotoxicity [50,51]. In previous studies, it has been shown that flavonoids such as baicalein usually produce some oligomeric species that are not toxic (13).

Therefore, we measured the flavonoid content of the extracts. Our analysis showed that among all extracts containing different degrees of flavonoids, DCMEx contained the higher amount of flavonoids (2/244 mg baicalein/g of dry material). In comparison with Hex and EtOAc based on solvent polarity, DCM, belongs to the partially polar solvent groups, with 9.1 dielectric constant and the potential to extract semi polar compounds such as flavonoids [52]. Furthermore, using TLC, HPLC and MS analysis we confirmed that there is a small amount of baicalein in the extracts, especially in DCMEx; however more experiments need to be done in order to confirm the data (e.g. NMR or IR analysis). Many flavonoids including baicalein and epigallocatechin gallate are well-known multifunctional neuroprotective compounds against misfolded-protein induced toxicity, especially α***-***SN [13,14]. It has also been proved that flavonoids are strong antioxidant components [23].

We observed a high correlation between the flavonoid content and the anti-fibrillation properties of the extracts. By comparing the results collected from fibril formation assessment with those obtained from the analysis of the flavonoids content, it seems that DCMEx, the extract with the highest amount of flavonoid, is also the extract with the highest anti-fibrillation effects. In contrast HexEx has the lowest flavonoid content and the least inhibitory effect on α***-***SN fibril formation. These findings suggest that the anti-fibrillation activity of DCMEx may be attributed mainly to its flavonoids compounds, in accordance with previous studies [16,53,54].

A critical event in PD is oxidative stress, which leads to the overproduction of ROS and is a common pathogenic mechanism in neurodegeneration [55]. ROS has a deleterious impact on vital cellular components, which cause cellular impairment and apoptosis [56]. Based on the known antioxidant properties of flavonoids, we tested the antioxidant activity of *S*. *pinnatifida* extracts using the DPPH assay. Our results show that MeOHEx and also its related sub-extracts have a high free radical scavenging activity with a strong correlation between the flavonoid content and the diminution of ROS production induced by α-SN. The results further showed that EtOAcEx and DCMEx were the most active extracts in scavenging DPPH radicals. It is reasonable to expect that higher level of flavonoids content leads to higher antioxidant activity [57,58].

In a next step we tested the effect of inhibiting α-SN-aggregation with the extracts on their cellular toxicity by comparing the effect of α-SN incubated alone or in the presence of the extracts on PC12 cells. Our results show that incubating α-SN with BuOHEx had a significant protective effect on cell toxicity when compared to the effect of 7h-aged α-SN alone, that lead to cell death in concordance with previous studies [59]. It seems that BuOHEx has compounds with the ability to prevent cytotoxicity of the aggregated α-SN in the primary stages of the fibrillation process. On the other hand, co-treatment with α-SN and 100 μg/mL DCMEx and its presence in the media lead to death of PC12 cells, suggesting that, at this concentration this extract is toxic for PC12 cells. This toxicity seems to be concentration dependent, and 1 μg/mL of DCMEx was not toxic for PC12 cells. Interestingly, we did co-treatment dopaminergic neurons with toxic oligomeric form of α-SN and 1 μg/mL DCMEx. At this concentration DCMEx did not inhibit the fibrillation process of α-SN properly, suggesting that mechanism of its protective against toxicity of α-SN is different. In this regards, further studies need to be done supplementary fractionation of the DCMEx to find a fraction with high anti-fibrillation effects but without any considerable neurotoxicity.

Recent studies have shown that extracellular α-SN aggregates can be transported to neurons and are mostly responsible for propagation of α-SN pathology[10]. We assumed that if the neurons contaminate with extracellular α-SN similar to the pathophysiological situation of PD, the extracts can help neurons against neurotoxicity of α-SN. We used a mixture of α-SN containing α-SN aggregated in the absence of the extracts to treat primary mesencephalic neuronal cultures containing dopaminergic neurons. In this experimental setup, we show that α-SN oligomers increase ROS production and lead to death of dopaminergic neurons. Adding BuOHEx/EtOAcEx (in a ratio of 1 to 1(v/v)) or DCMEx to the media reduced α-SN-oligomers cytotoxicity and ROS production. Thus suggesting that the extracts were able to penetrate neurons and exert their effect intracellularly. Moreover, in order to analyze the anti-oxidant properties of the extracts alone, we treated dopaminergic neurons with the pesticides paraquat and rotenone. Paraquat induces NADPH depletion and ROS production in the cell and rotenone is an inhibitor of the mitochondrial Complex I. Interestingly, our results show that both BuOHEx/EtOAcEx and DCMEx were able to reduce the toxicity of paraquat but not rotenone, suggesting a protective effect against ROS production but not against mitochondrial function impairment. Overall our results suggest that the neuroprotective effect of DCMEx and BuOHEx/EtOAcEx is due to a double effect inhibiting α-SN-aggregation and protecting against ROS. Additionally, two important flavonoids, wogonin and skullcap flavone II, which have been identified in the roots of *S*. *pinnatifida* [60,61] exhibit significant anti-inflammatory effects [26,62]. The anti-inflammatory activity of wogonin is connected with its ability to inhibit *NF-κB* pathway [26–28]. Evidence shows that scullcap flavones exert potent anti-inflammatory effects by preventing the expression of monocyte chemotactic protein-1, a main factor for the early inflammatory responses [63,64]. We speculate that this effect may play an additional role in the neuroprotective effect of the extracts, but this hypothesis needs to be further evaluated.

There is no prior study on the antioxidant properties of *S*. *pinnatifida*. High level of the flavonoids in the extracts supports the importance of this plant as an excellent herbal source of antioxidants and amyloid inhibitors. It seems obvious that the constituents present in the extracts, such as flavonoids and especially baicalein, may have important roles for this activity. However, further research needs to be done in order to explore the chemical constituents present in the extracts, which are responsible for the anti-fibrillation activity.

## Supporting information

S1 Fig Generation of recombinant human α*-*SN oligomers. 2D-FIDA Histogram showing the size distribution of Alexa Fluor-647 tagged α-SN incubated in the absence (**A**) or presence (**B**) of 100 µM Al^3+^.
(TIF)

S2 Fig HPLC analysis of DCMEx compared with baicalein standard. (PSD)

S3 Fig Ion mass and MRM transition ion mass spectra of baicalein (A, B) and DCMEx (C, D).
(PSD)

S4 Fig Treatment of PC12 with α-SN pretreated with 1, 10, 100 µg/mL of DCMEx and 100 µg/mL of MeOHEx or BuOHEx. (PSD)

S1 Table Variation in CM of CR absorbance spectrum in the presence of α-SN alone and in the presence of MeOHEx, HexEx, DCMEx, EtOAcEx, and BuOHEx. (DOCX)
