# Supplementary figures and images for: A study on the modulation of alpha-synuclein fibrillation by Scutellaria pinnatifida extracts and its neuroprotective properties
Source: PLoS One. 2017 Sep 28;12(9):e0184483. doi: 10.1371/journal.pone.0184483 (PMC5619708; doi:10.1371/journal.pone.0184483)

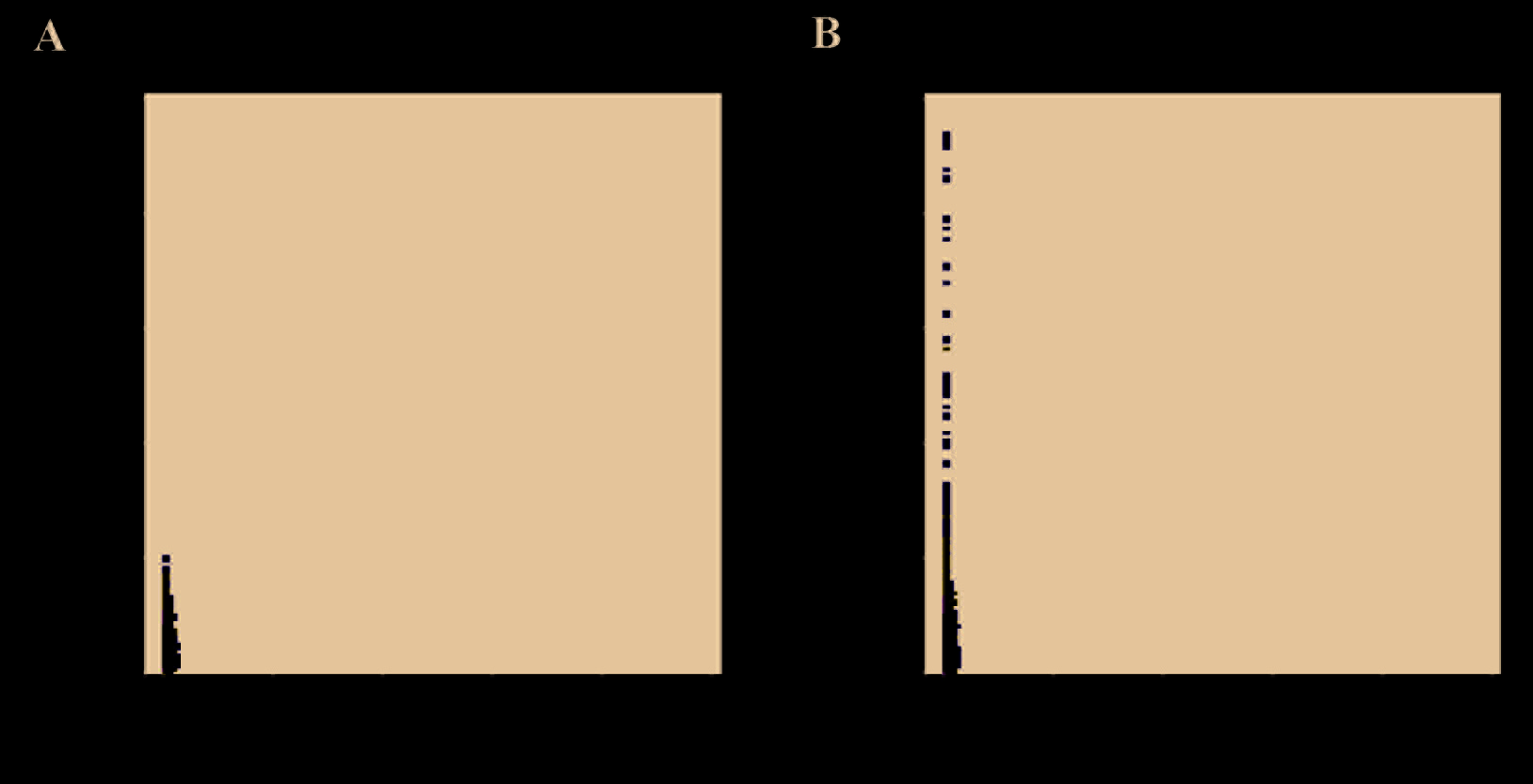

Supplement: S1 Fig — 2D-FIDA Histogram showing the size distribution of Alexa Fluor-647 tagged α-SN incubated in the absence (A) or presence (B) of 100 µM Al3+. (TIF) [file pone.0184483.s001.tif]
