# Supplementary material for: A study on the modulation of alpha-synuclein fibrillation by Scutellaria pinnatifida extracts and its neuroprotective properties
Source: PLoS One. 2017 Sep 28;12(9):e0184483. doi: 10.1371/journal.pone.0184483 (PMC5619708; doi:10.1371/journal.pone.0184483)
Supplement: S1 Table — (DOCX) [file pone.0184483.s005.docx]

S1 Table. Variation in CM of CR absorbance spectrum in the presence of α-SN alone and in the presence of MeOHEx, HexEx, DCMEx, EtOAcEx, and BuOHEx.

| **Sample** | CM |
| --- | --- |
| CR | 497.7693 |
| + α-SN | 514.1837 |
| + α-SN +Met | 502.4299 |
| + α-SN +HexEx | 513.6935 |
| + α-SN + DCMEx | 503.513 |
| + α-SN + BuOHEx | 502.7238 |
| + α-SN + EtOAcEx | 505.3164 |
